# ZeOxaNMulti Trial: A Randomized, Double-Blinded, Placebo-Controlled Trial of Oral PMA-Zeolite to Prevent Chemotherapy-Induced Side Effects, in Particular, Peripheral Neuropathy

**DOI:** 10.3390/molecules25102297

**Published:** 2020-05-13

**Authors:** Maria Giuseppa Vitale, Carmela Barbato, Anna Crispo, Francesco Habetswallner, Bernardo Maria De Martino, Ferdinando Riccardi, Angela Maione, Sandra Eisenwagen, Giovanna Vitale, Giacomo Cartenì

**Affiliations:** 1 Medical Oncology Unit, University Hospital of Modena, 41125 Modena, Italy; 2 Medical Oncology Unit, AORN Antonio Cardarelli, 80131 Naples, Italy; carmelabarbato67@gmail.com (C.B.); nando.riccardi@gmail.com (F.R.); angela_maione@live.it (A.M.); cartenigiacomo@gmail.com (G.C.); 3 Epidemiology and Biostatistics Unit, Istituto Nazionale Tumori-IRCCS “Fondazione G. Pascale”, 80131 Naples, Italy; a.crispo@istitutotumori.na.it; 4 UOC Neurofisiopatologia AORN Cardarelli, 80131 Naples, Italy; francesco.habetswallner@fastwebnet.it (F.H.); bernardodemartino@alice.it (B.M.D.M.); 5 Panaceo International GmbH, 9585 Goedersdorf, Austria; sandra.eisenwagen@panaceo.com; 6 School of Medicine and Surgery, University of Campania Luigi Vanvitelli, 81100 Caserta, Italy; vitalegiovanna95@gmail.com

**Keywords:** oxaliplatin, PMA-zeolite (clinoptilolite), chemotherapy-induced peripheral neuropathy, liver toxicity, hematological toxicity, cancer

## Abstract

Chemotherapy-induced peripheral neuropathy (CIPN) is the most frequently reported adverse effect of oxaliplatin. In this study, we set out to evaluate the role of the panaceo-micro-activation (PMA) zeolite in the reduction of the incidence of CIPN and hematological and liver toxicity. The possible impact of the PMA-zeolite as an adjuvant therapeutic agent is based on its detoxification properties toward agents promoting the development of neuropathy (e.g., ammonium—recognized as a neurotoxic agent produced by tumors), as well as its positive impact on immunity and oxidative stress through its effects in the gastrointestinal tract. From April 2015 to October 2018, a total of 120 patients (pts) diagnosed with predominantly colorectal cancer requiring oxaliplatin-based chemotherapy were randomized to receive either the PMA-zeolite (Multizeo Med) or placebo while undergoing oxaliplatin-based chemotherapy. A nerve-conduction study (NCS) was planned at the baseline, after three and six months of chemotherapy, to evaluate CIPN. Furthermore, the evaluation of hematological and liver toxicity was performed during every cycle of chemotherapy. 70.6% and 64.3% of patients developed CIPN in the placebo and the PMA-zeolite group, respectively. Patients treated with the PMA-zeolite were able to undergo more cycles of chemotherapy (*p* = 0.03), which also indicates a significant improvement in tolerance to the therapy. The group treated with the PMA-zeolite showed a lower CIPN (although not statistically significant within the whole group of subjects) compared to patients receiving placebo. This advantage was, however, statistically significant in men (*p* = 0.047). In addition, supplementation with the PMA-zeolite resulted in a lower incidence of severe-grade hematological toxicity (trend toward statistical significance of *p* = 0.09 was observed). Cancer patients may benefit from the therapy with the appropriate certified zeolite-products (e.g., the PMA-zeolite) for human use in CIPN. The lower CIPN (statistically significant results in the male subgroup) was accompanied by a trend of lower incidence of severe-grade hematological toxicity. Furthermore, these benefits led to a better tolerance toward chemotherapy (increase in cycles) and allow an improved compliance with the oncological treatment protocol.

## 1. Background

The development of chemotherapy-induced peripheral neuropathy (CIPN) during antineoplastic therapy is one of the most common causes of the termination or modification of cancer treatment. Toxicity results from the inability of chemotherapeutic agents to differentiate between healthy and malignant cells, which causes significant patient morbidity.

The type and degree of injury to the peripheral nerves are variable and dependent on several factors, including the specific antineoplastic agent, duration of therapy, and cumulative dose. The cytotoxic drugs most frequently responsible for neurotoxic symptoms are anti-microtubule agents (vinca alkaloids and taxanes) and platinum derivatives (cisplatin, carboplatin, oxaliplatin) [1].

Platinum-induced peripheral neuropathy may cause pain and function loss and can be a dose-limiting factor for cancer treatment. In a systematic review of 31 studies (N = 4179), CIPN prevalence had a wide variance from 12.1% to 96.2%, depending on different timings of assessment and type of chemotherapy [2]. The signs and symptoms of reversible acute neuropathy were furthermore observed in 82% to 98% of patients in five clinical trials involving 210 patients [3].

The most significant adverse reaction associated with the use of oxaliplatin is CIPN with both acute and cumulative symptoms. Acute transient neuropathy, which can appear during or shortly after the first few infusions and usually manifests itself as distal and/or perioral dysesthesia, is determined in approximately 85% to 95% of patients receiving oxaliplatin. These sensory symptoms, which are triggered by exposure to cold, tend to resolve spontaneously within a few hours or days. The cumulative peripheral neuropathy, which is characterized by a paraesthesia or dysesthesia functional impairment of the extremities, constitutes the dose-limiting toxicity of oxaliplatin [4,5,6,7]. Chronic CIPN is due to platinum accumulation in the dorsal root ganglion sensorial and, in particular, to the inhibition of the synthesis of rRNA in the nucleoli of neurons, leading to a morphological change and damage of sensorial neurons [8].

Grothey and Cersosimo reported an incidence of chronic neuropathy (grade 3/4) of 16% [5,9].

According to ASCO Guidelines, there is no treatment of proven efficacy for CIPN prevention, and serotonin-norepinephrine reuptake inhibitor (SNRI) duloxetine is the only drug proven to be effective [10].

The distress of the affected population can be quite high, such as when toxins cause neuropathies. In chemotherapy, the toxic and, therefore, neuropathic side effects can be quite high due to the inability of chemotherapeutic agents to differentiate between healthy and malignant cells. In this context, zeolites (clinoptilolite materials) might be an option as adjuvant substances in cancer therapy due to these materials’ specific properties. Zeolites (clinoptilolite materials) are naturally occurring aluminosilicates originating mainly from volcanic origin. They have a microporous network forming small individual channels and cavities possessing cation-exchange capacity. Based on their absorbency and ion-exchange capacity, they are used in a broad field of applications, including industrial, zootechnical, or biotechnological fields, as well as in human medicine. Recently, the application of a specific natural zeolite material, the panaceo-micro-activation (PMA)-zeolite (clinoptilolite material), has proven to be an efficient and safe option for many medical purposes. The PMA-zeolite (clinoptilolite material) is a certified medical device for human use and its detoxifying, antioxidant, and anti-inflammatory properties are based on its porous structure with the ability of capturing ions and molecules into its holes [11]. This mechanism of action is quite specific as the negatively charged channels and cavities are occupied with positively charged alkali, and alkali earth monovalent (i.e., Na^+^, K^+^) and divalent (i.e., Ca^2+^) ions, OH-groups, or H_2_O molecules. These molecules are readily exchangeable through other elements and cations from the surrounding environment. Another essential issue that determines the ion exchange capacity and attraction of cations is the final Si/Al ratio in the zeolite (clinoptilolite material) [12,13].

It should be emphasized that not every zeolite is safe for usage in human applications. Some zeolites are synthesized in the laboratory as artificial materials. Those, such as zeolite A, may not be suitable for in vivo applications. Zeolite A is indeed unstable in acids and, therefore, in contrast to natural occurring zeolites (clinoptilolites), unsafe for application in humans [12].

The positive systemic mechanisms of natural zeolites (clinoptilolite materials) are still not completely understood. Pavelić et al. hypothesize that they may be at least partially attributed to the restoration of the human homeostasis due to local detoxification properties within the intestine, the release of dissolved silica forms from the clinoptilolite tuff that enters from the intestine into the blood, as well as to clinoptilolite’s immunomodulatory effects involving the induction of immune responses through Peyer’s patches and/or possible positive effects on microbial intestinal populations. These local effects may strengthen the whole immune system [12].

The acute CIPN, induced by oxaliplatin, is due to the temporary interaction with voltage-gated sodium channels (Na^+^) in the nerve membrane. In that regard, the PMA-zeolite might have a positive impact due to the described porous structure containing cavities that are preloaded with cations, such as sodium (Na^+^). As Na^+^ is readily exchangeable [13], its absorption in vivo is not expected to be decreased; however, the opposite may occur. Along with the release of positively charged minerals, other molecules and cationic groups from the surroundings, such as ammonia, can be accommodated inside the porous structure [14,15]. The cations are bound to the zeolite (clinoptilolite material) based on their selectivity alignment [12]. Besides ammonia, which is generally known as a neurotoxin and produced by tumors or during chemotherapy, numerous heavy metals, such as Pb, As, Cr, Ni or Cd, may be exchanged under the physiological conditions of the gastrointestinal tract [16]. However, the zeolite (clinoptilolite material) ion-exchange effects in vivo are complex and depend on both the environmental conditions (pH, temperature, etc.) and material composition/cation affinity properties and are, therefore, not linearly explainable.

At present, studies suggest a correlation between a healthy state of the gut and the diversity of gut bacteria, in particular, for a proper response to cancer therapy—specifically in the case of immunotherapy [17].

In a recent review article, it was summarized by Pavelić et al. that the positive immunomodulatory effects of zeolite (clinoptilolite material) may be due to the interactions of particles in the intestine with microfold cells (M-cells), which are found in the Gut-Associated Lymphoid Tissue (GALT) of Peyer’s patches. They are a rich lymphoid tissue communicating with intestinal epithelial cells and the microbiome of the intestine through various immunomodulation processes. These gastrointestinal cells are known to initiate mucosal immunity responses on the apical membrane of the M-cells and to allow the transport of microbes and particles across the epithelial cell layer from the gut lumen to the lamina propria, where interactions with immune cells occur [12,18]. The immunomodulatory effects of activated zeolite (clinoptilolite material) may be due to interactions with M cells [19]. Indeed, M cells could interact with the zeolite (clinoptilolite), which could, then, induce changes in the redox homeostasis and affect Peyer’s patches. These absorbed microparticles do not pass to the bloodstream but rather act locally in this district, as hypothesized by Lamprecht and colleagues. They noticed a positive impact on IL-10 and, consequently, on induction of anti-inflammatory processes in the intestinal lymphoid tissues [20].

Preclinical studies in farmed poultry showed a positive effect of a natural zeolite (clinoptilolite material) on balancing the total intestinal microbial flora, reducing toxic effects of aflatoxins and increasing the antioxidant activity of peroxidase, catalase, and superoxide dismutase (SOD) [21,22,23,24]. This supporting effect on the body’s own antioxidative mechanisms is a further hypothesis for the observed clinoptilolite immunomodulatory effects. This might be well connected to the body’s defense mechanisms against reactive oxygen species (ROS). Indeed, ROS induces cell and tissue damage when the inflammation is initiated as a mechanism for the restoration of the body’s homeostasis. Although a controlled production of ROS is essential for the body’s homeostasis [25], an excessive ROS causes oxidative damage to the DNA, proteins, and lipids [26]. Modulation of the ROS-regulated signaling pathways and oxidative damage to cells and tissues is related to the pathogenesis of a wide number of diseases, including atherosclerosis, hypertension, neurological diseases, and cancer [12]. In this regard, a preliminary efficacy study with patients suffering from dyslipidemia and orally supplementing a zeolite (clinoptilolite material) lowered the total lipid count and low-density lipoproteins (LDL)—this might also be indirectly correlated with the general antioxidative effect [27].

Due to a certain amount of pre-loaded elements, it is possible to assume that clinoptilolite may positively affect the body’s metal homeostasis, including either the levels or the availability of some physiological metal ions, which are pre-loaded in the material and on the signaling pathways responsible for the production of endogenous antioxidant enzymes. Zeolite (clinoptilolite material), in addition, shows whitening, hemostatic, and anti-diarrheic properties projected in human application [28,29].

As data from various publications show, zeolite (clinoptilolite) materials are known to have positive effects on human health; however, not every clinoptilolite is adequate for human use due to a lack of data related to the specific clinoptilolite materials safety and efficacy [13]. These data are, however, available for the specific PMA-zeolite (clinoptilolite) (Multizeo Med) used in the presented study.

In the field of cancer research, some preclinical studies suggest a positive impact of the zeolite (clinoptilolite material) so far [30,31,32]. In the context of oxaliplatin chemotherapy, the application of the zeolite (clinoptilolite material) might have a positive impact, as oxaliplatin is known to chelate extracellular calcium ions and decrease the action potential threshold to induce pain. This hypothesis is based on the study where zeolite (clinoptilolite material) has reduced the functionality of the key proteins involved in cell survival and apoptosis in tumor cell lines. This impact on tumor cell lines has been attributed to the absorptive and ion-exchange capacities that are able to modify the concentration of biomolecules and/or calcium ions and affect the Ca-dependent molecular signals [33].

Even though some studies have already suggested a positive impact of the natural zeolite (clinoptilolite material), only few clinical studies have been conducted so far in the field of oncology following chemotherapeutic treatment.

Based on these data, the aim of our study was to determine whether the PMA-zeolite might be useful in the prevention of chemotherapy-induced side effects, especially peripheral neuropathy for patients receiving oxaliplatin-containing chemotherapy.

## 2. Patients and Methods

### 2.1. Patients

From April 2015 to October 2018, 120 patients from the Oncology Department, Antonio Cardarelli Hospital, Naples, Italy were enrolled and screened for eligibility. Inclusion criteria were a histologically confirmed diagnosis of cancer, at least 18 years of age, oxaliplatin chemotherapy, and adequate hematologic parameters to allow chemotherapy. Only patients treated with oxaliplatin as a neurotoxic cytostatic were included.

Exclusion criteria were chemotherapy treatment with neurotoxic drugs (cis-platin, carboplatin, oxaliplatin, vincristine, vinblastine, paclitaxel, or docetaxel) in the six months prior to the start of oxaliplatin-chemotherapy, pregnancy, or breastfeeding. All participants gave their written informed consent. Eligible patients were randomized 1:1 in two groups: PMA-zeolite and placebo group. The study was approved by the ethical standards of the Antonio Cardarelli Hospital Ethical Committee (protocol number 107 of 19/02/2015). This study was conducted according to the guidelines of the Declaration of Helsinki for Research on Human Subjects 1989.

### 2.2. Study Design

This was a randomized, double-blind, placebo-controlled study. Patients treated with oxaliplatin-based chemotherapy (adjuvant, first or second line) that had agreed to participate in the study were randomized into blocks of four and sequentially numbered. The randomization code was held by a third party and handed over for statistical analyses after the collection of all data. The supplementation was carried out according to the following scheme: Experimental group received 6 g/day of the PMA-zeolite (Multizeo Med, Goedersdorf, Austria) in two daily doses of 3 g, while the control group received 6 g/day of placebo (microcrystalline cellulose as it was used in another randomized, double-blinded, placebo-controlled trial) [20] in two daily doses of 3 g. The placebo and experimental treatments were indistinguishable in size, weight, and their characteristics. Treatment with the PMA-zeolite/placebo started simultaneously at the beginning of the chemotherapy, considering the time interval to other drugs, and suspended for two days before and after the administration of chemotherapy. The treatment was practiced throughout the duration of chemotherapy and continued for a month after chemotherapy had terminated. Patients were treated until disease progression, unacceptable toxicity, or the patient’s refusal to continue the treatment.

The primary objective was the reduction of the acute CIPN in patients treated with oxaliplatin. Assuming an incidence of the acute CIPN of 85% among patients treated with oxaliplatin-based chemotherapy, our study has a power of 86% to show that the acute CIPN incidence rate in the PMA-zeolite group is 75%. Assuming that the difference of 10 points or less between the two groups and its 95% confidence interval, as well as that alpha (2-tailed) is set at 0.05, the sample size was two groups comprising 60 patients each. Formally, the likelihood was 86.5% that the 95% confidence interval for the difference in event rates will exclude a 10% point difference in favor of the placebo group.

The second objective was to estimate the reduction of hematological and liver toxicity induced by chemotherapy.

### 2.3. Chemotherapy-Induced Peripheral Neuropathy, Hematological- and Hepatological Toxicity

The patients were examined by two neurologists. The neurologists performed neurological examination and electroneurographical tests [34,35,36]. Sensory nerve action potential amplitudes and conduction velocities were measured in the bilateral peroneal and sural nerves. Compound motor action potential amplitudes and motor nerve conduction velocities were evaluated in the common peroneal and tibial nerves. A nerve conduction study (NCS) was planned before the start of oxaliplatin-chemotherapy (Neurography 1) after three months (Neurography 2) and six months (Neurography 3) to evaluate the CIPN.

The grading of the CIPN level included: Normal, mild, moderate, and severe; the variable was categorized into normal; mild + moderate; severe, and as a response variable to the presence or absence of CIPN.

Hematological (red blood cells, hemoglobin, white blood cells, and platelets) and liver (AST, ALT, total and fractionated bilirubin, Gamma-GT) toxicities were analyzed in every cycle of chemotherapy. The grading of hematological and liver toxicities was expressed in four grades according to Common Terminology Criteria for Adverse Events (CTCAE), version 4.03: G1, G2, G3, and G4.

### 2.4. Statistical Analysis

Descriptive statistics for the categorical data were reported. Associations between categorical variables were evaluated using the chi-squared test. Relative risk was performed with the CIPN as the response variable and the treatment group. Multivariate logistic models were utilized to evaluate crude and adjusted odds ratios (ORs), and 95% confidence intervals (95% CIs) for all possible confounding factors, including age, gender, number of chemotherapy cycles received, and comorbidity. All statistical tests were two-sided. *p*-values < 0.05 were considered significant. Statistical analyses were performed using the Statistical Package for Social Science (SPSS), statistical software version 25 (SPSS inc., Chicago, IL, USA).

## 3. Results

### 3.1. Demographic Characteristics

From April 2015 to October 2018, 120 patients (pts) with colorectal cancer (112 pts, 93.3%) or other cancer types (8 pts, 6.7%) from the Oncology Department, Antonio Cardarelli Hospital, Naples, Italy were enrolled and screened for eligibility.

The majority of patients were males (55%) and under 60 years of age (40.8%). The most frequent comorbidities were diabetes and hypertension.

In addition, 88.8% of patients received the FOLFOX scheme (oxaliplatin plus leucovorin and 5-fluorouracil) and 10% the XELOX scheme (capecitabine plus oxaliplatin). Only 5% of patients discontinued the ongoing chemotherapy treatment, while in 50%, the oxaliplatin dose had to be reduced.

Then, 70% of patients had “normal” neurography before the start of chemotherapy (“Neurotoxicity 1”), while the incidence of “normal” neurography performed after the end of chemotherapy was reduced to only 20.8% (“Neurotoxicity 3”). The incidence of hematologic and liver toxicity was 45% and 5%, respectively. Detailed characteristics for the whole sample are shown in Table 1.

### 3.2. Outcomes

The outcome variables were “Neurography 1” and “Neurography 3;” the response rates were 94.2% and 64%, respectively (Table 2). They were categorized into a binary variable (presence/absence of chemotherapy-induced peripheral neuropathy (CIPN)), in particular, “normal” = 0 (absence of CIPN) and “abnormal: Mild/moderate/severe” = 1 (presence of CIPN).

Table 2 shows the associations, including the group variable (placebo/PMA-zeolite): A statistically significant difference was observed with age (*p* = 0.05), where, in the PMA-zeolite group, 50% were below the age of 60, and only 18.3% exceeded the age of 70 y. In addition, 71.7% of treated patients received more than eight cycles of chemotherapy against 56.7% of the placebo group (*p* = 0.03), and 56.7% of the treated group had no comorbidity compared to 35% of the placebo group (*p* = 0.017).

The incidence of CIPN observed before the start of chemotherapy (Neurography 1) was 25% in the control group (placebo) and 26.3% in the intervention group (PMA-zeolite) (response rate 94.2%); this difference was not statistically significant (*p* = 0.87). After six months from the last cycle of chemotherapy, the incidence of the CIPN (Neurography 3) in the placebo group was higher than that in the PMA-zeolite group (70.6% and 64.3%, respectively), although this difference did not reach statistical significance (*p* = 0.56) (response rate 64%).

Table 3 shows the association between the outcome (Neurotoxicity 3) and the whole cohort (placebo/PMA-zeolite) for the calculation of the relative risk (RR = 0.91). The logistic regression analysis calculated the odds ratio (unadjusted) (OR = 0.75; 95% CI 0.28–1.98) and adjusted to age, sex, cycles of chemotherapy, and comorbidity (OR = 0.66; 95% CI 0.23–1.91). The decreasing risks (from 0.91 to 0.66) showed an advantage for the group treated by neurotoxic events even if the significance was not reached.

When the analysis was performed according to sex (Table 4), it was observed that fewer CIPN events decreased in men in the treated group (PMA-zeolite group) and increased with the incidence of 68.2% vs. 94.1% in the untreated group (placebo group); this difference was statistically significant (*p* = 0.047).

The incidence of hematological toxicity was 43.1% in the placebo group and 49.2% in the PMA-zeolite group. The association with hematological toxicity was not statistically significant (*p* = 0.09), but a trend toward severe toxicity (G2-3) was observed in the placebo group by 12.1% as opposed to 3.4% in the PMA-zeolite group (Table 5).

In view of the low incidence of liver toxicity reported in our trial, we did not perform a corresponding statistical analysis.

## 4. Discussion

To the best of our knowledge, this is the first randomized, prospective-controlled clinical study to evaluate the role of the PMA-zeolite (Multizeo Med) in oxaliplatin-induced peripheral neuropathy in cancer patients.

CIPN is a common and dose-limiting toxicity that negatively impacts both the disease outcomes and quality of life. To date, there is no treatment with proven efficacy for CIPN. Although more than 40 randomized trials have evaluated a variety of pharmacologic interventions for the treatment of CIPN, only duloxetine, a serotonin and norepinephrine reuptake inhibitor, has shown clear efficacy in the phase III study [11,37,38,39].

The cumulative dose of neurotoxic drugs represents the most important risk factor for CIPN [39,40,41]. Other important triggers of CIPN include prior or concomitant administration of different neurotoxic drugs (such as cisplatin plus paclitaxel combination therapy); pre-existing peripheral neuropathy (e.g., advanced age, diabetes complications, alcoholic neuropathy, and other concomitant medications); the severity of acute chemotherapy-induced peripheral neuropathy (in the case of oxaliplatin); and short infusion times: 2 versus 4 or 6 h for oxaliplatin infusions [2,42].

Although our study did not achieve the pre-planned primary and secondary objectives, it allowed us to obtain important data. Neurotoxicity remains a significant problem at the end of chemotherapy, and only 20% of patients have no alterations detected on neurography compared to 70% of the baseline.

In the present study, patients treated with the PMA-zeolite showed lower CIPN compared to the patients treated with placebo, although the statistical significance was not reached. Furthermore, when this result was further analyzed with regard to sex (Table 4), fewer CIPN events were detected in men with an incidence of 68.2% and 94.1% in the treated group (PMA-zeolite) and the placebo group, respectively. This means that sex-related analysis of the data revealed a statistically significant lower CIPN in the male subgroup (*p* = 0.047). One hypothesis for observed sex-related differences in obtained results might be due to the differences in oxidative stress mechanisms. Under physiological conditions, females appear to be less susceptible to oxidative stress. This may be due to the antioxidant properties of estrogen, sex differences in NADPH-oxidase activity, or other mechanisms yet to be studied in more details. This effect was nevertheless already studied in a wide range of cardiovascular diseases [43]. In addition, the ROS levels may partially explain the observed differences. For example, the results from previous studies indicate that increased ROS levels in the spinal cord may induce pain by reducing GABA (gamma-Aminobutyric acid) inhibitory influence on SG neurons that are involved in pain transmission [44].

As neuropathy pain could, therefore, be a consequence of the imbalance in ROS and endogen antioxidants mechanisms, the sex difference in ROS mechanisms may underlie different results between male and female subjects in the study presented herein.

We have also noticed that the correlation between electrophysiological measurement and the presence of neurological symptoms was not always consistent. Sometimes, neurography was already slightly altered in the absence of symptoms reported by the patients.

We also observed that patients treated with the PMA-zeolite were able to undergo more cycles of chemotherapy; this difference was statistically significant (*p* = 0.03). This may be interpreted as a clinically positive result as it points to a significantly improved compliance toward standard therapy protocol.

The PMA-zeolite was also connected with a lower incidence of severe hematological toxicity. Even though the results in the study were not statistically significant (*p* = 0.09), a more severe toxicity trend (G2-3) was observed in the placebo group (12.1% vs. 3.4% in the PMA-zeolite group).

Furthermore, the treatment with the PMA-zeolite was well-tolerated without any unexpected toxicity.

The results obtained in the study presented herein are encouraging for the adjuvant usage of the PMA-zeolite in chemotherapy protocols.

## 5. Conclusions

Oxaliplatin-based chemotherapy substantially deteriorates the neurologic condition of patients and the quality of life. Patients may benefit from adjuvant therapy with appropriate zeolite-products (e.g., PMA-zeolite) in CIPN. A lower level of CIPN was observed in the cohort tested herein in general, and particularly in the male subgroup, statistically significant results were obtained. Furthermore, better tolerance toward chemotherapy (increased number of cycles) accompanied by a lower incidence of severe-grade hematologic toxicity was achieved.

## Figures and Tables

**Table 1:** Sample characteristics.

	N = 120	%
**Sex**		
Male	66	(55.0)
Female	54	(45.0)
**Age**		
<60	49	(40.8)
61–65	17	(14.2)
66–70	29	(24.2)
>70	25	(20.8)
**BMI**		
<24.9	72	(60.0)
25.0–29.9	40	(33.3)
≥30.0	8	(6.7)
**Type of cancer**		
Colorectal	112	(93.3)
Other	8	(6.7)
**Chemotherapy scheme**		
Folfox	106	(88.3)
Xelox	12	(10.0)
Other	2	(1.7)
**Chemotherapy line**		
Adjuvant	49	(40.8)
First line	60	(50.0)
Second Line	7	(5.8)
Third Line	3	(2.5)
Unknown	1	(0.8)
**Number of chemotherapy cycles received**		
≤6 cycles	23	(19.2)
7–8 cycles	20	(16.7)
>8 cycles	77	(64.2)
**Comorbidity**		
No	55	(45.8)
Yes	65	(54.2)
**Suspension Chemotherapy**		
No	110	(91.6)
Yes	6	(5.0)
Unknown	5	(3.3)
**Reduction Oxaliplatin dose**		
No	56	(46.7
Yes	60	(50.0)
Unknown	4	(3.3)
Neurotoxicity of Neurography * 1		
Normal	84	(70.0)
Mild	22	(18.3)
Moderate	6	(5.0)
Severe	1	(0.8)
Unknown	7	(5.8)
Neurotoxicity of Neurography * 2		
Normal	47	(39.2)
Mild	24	(20.0)
Moderate	7	(5.8)
Severe	2	(1.7)
Unknown	40	(33.3)
Neurotoxicity of Neurography * 3		
Normal	25	(20.8)
Mild	30	(25.0)
Moderate	20	(16.7)
Severe	1	(0.8)
Unknown	44	(36.7)
**Hematological toxicity**		
No	63	(52.5)
G1	45	(37.5)
G2	7	(5.8)
G3	2	(1.7)
Unknown	3	(2.5)
**Liver toxicity**		
No	111	(92.5)
G1	5	(4.2)
G2	1	(0.8)
Unknown	3	(2.5)

* Neurotoxicity 1: CIPN at the baseline; Neurotoxicity 2: CIPN after 3 months; Neurotoxicity 3: CIPN after 6 months.

**Table 2:** Distribution of demographic and clinical characteristics.

	Control Arm (Placebo) N = 60 (%)	Intervention Arm (PMA-Zeolite) N = 60 (%)	*p*-Value *
**Sex**			0.46
Male	35 (58.3)	31 (51.7)	
Female	25 (41.7)	29 (48.3)	
**Age**			0.054
<60	19 (31.7)	30 (50.0)	
61–65	13 (21.7)	4 (6.7)	
66–70	14 (23.3)	15 (25.0)	
>70	14 (23.3)	11 (18.3)	
**BMI**			0.44
<24.9	38 (63.3)	34 (56.7)	
25.0–29.9	17 (28.3)	23 (38.3)	
≥30.0	5 (8.3)	3 (5.0)	
**Type of cancer**			0.46
Colorectal	55 (91.7)	57 (95.0)	
Other	5 (8.3)	3 (5.0)	
**Chemotherapy scheme**			0.47
Folfox	51 (85.0)	55 (91.7)	
Xelox	8 (13.3)	4 (6.7)	
Altro	1 (1.7)	1 (1.7)	
**Chemotherapy line**	**(n = 60)**	**(n = 59)**	0.36
Adjuvant	24 (40.0)	25 (42.4)	
First line	30 (50.0)	30 (50.8)	
Second Line	3 (5.0)	4 (6.8)	
Third Line	3 (5.0)	0	
**N. chemotherapy cycles received**			0.03
≤6 cycles	17 (28.3)	6 (10.0)	
7–8 cycles	9 (15.0)	11 (18.3)	
>8 cycles	34 (56.7)	43 (71.7)	
**Comorbidity**			0.017
No	21 (35.0)	34 (56.7)	
Yes	39 (65.0)	26 (43.3)	
**Suspension Chemotherapy**	**(n = 57)**	**(n = 59)**	0.96
No	54 (94.7)	56 (94.9)	
Yes	3 (5.3)	3 (5.1)	
**Reduction Oxaliplatin dose**	**(n = 57)**	**(n = 59)**	0.19
No	31 (54.4)	25 (42.4)	
Yes	26 (45.6)	34 (57.6)	
**Neurography 1**	**(n = 56)**	**(n = 57)**	0.60
**(94.2% Response rate)**			
Normal	42 (75.0)	42 (73.7)	
Mild + Moderate	14 (25.0)	14 (24.6)	
Severe	0	1 (1.8)	
**Neurography 3**	**(n = 34)**	**(n = 42)**	0.47
**(64% Response rate)**			
Normal	10 (29.4)	15 (35.7)	
Mild + Moderate	23 (67.6)	27 (64.3)	
Severe	1 (2.9)	0	
**Neurography 1**	**(n = 56)**	**(n = 57)**	0.87
No “Neurotox”	42 (75.0)	42 (73.7)	
Yes “Neurotox”	14 (25.0)	15 (26.3)	
**Neurography 3**	**(n = 34)**	**(n = 42)**	0.56
No “Neurotox”	10 (29.4)	15 (35.7)	
Yes “Neurotox”	24 (70.6)	27 (64.3)	

* Chi-square test.

**Table 3:** Univariate and multivariate analysis.

	Neurotoxicity 3			RR	
	Yes (%)	No	Total	(95% CI)	
**Intervention Arm (PMA-zeolite)**	27 (64.3)	15	42	0.91 (0.28–1.98)	
**Control Arm (Placebo)**	24 (70.6)	10	34		
**Total**	51	25	76		
				**Crude OR** **(95% CI)**	Adjusted OR * **(95% CI)**
**Intervention Arm (PMA- zeolite)**				0.75 (0.28–1.98)	0.66 (0.23–1.91)
**Control Arm (Placebo)**					
***p*-value**				0.5	0.4

* Adjusted to age, sex, cycle, comorbidity.

**Table 4:** Chemotherapy-induced peripheral neuropathy (CIPN) univariate analysis stratified by gender.

	Control Arm (Placebo) N (%)	Intervention Arm (PMA-Zeolite) N (%)	*p*-Value
**MALE = 39**			
**Neurotoxicity 3**			0.047
No	1 (5.9)	7 (31.8)	
Yes	16 (94.1)	15(68.2)	
**FEMALE = 37**			
**Neurotoxicity 3**			0.43
No	9 (52.9)	8 (40.0)	
Yes	8 (47.1)	12 (60.0)	

**Table 5:** Hematologic toxicity—univariate analysis.

	GROUP		
	Control Arm (Placebo) N = 58	Intervention Arm (PMA-Zeolite) N = 59	*p*-Value *
**Hematological toxicity**			0.09
No	33 (56.9)	30 (50.8)	
Grade 1	18 (31.0)	27 (45.8)	
Grade 2—Grade 3	7 (12.1)	2 (3.4)	

* Chi-square test.
